# Structure of HsdS Subunit from *Thermoanaerobacter tengcongensis* Sheds Lights on Mechanism of Dynamic Opening and Closing of Type I Methyltransferase

**DOI:** 10.1371/journal.pone.0017346

**Published:** 2011-03-02

**Authors:** Pu Gao, Qun Tang, XiaoMin An, XiaoXue Yan, DongCai Liang

**Affiliations:** 1 National Laboratory of Biomacromolecules, Institute of Biophysics, Chinese Academy of Sciences, Beijing, China; 2 Graduate University of Chinese Academy of Sciences, Beijing, China; University of Washington, United States of America

## Abstract

Type I DNA methyltransferases contain one specificity subunit (HsdS) and two modification subunits (HsdM). The electron microscopy model of M.EcoKI-M_2_S_1_ methyltransferase shows a reasonable closed state of this clamp-like enzyme, but the structure of the open state is still unclear. The 1.95 Å crystal structure of the specificity subunit from *Thermoanaerobacter tengcongensis* (TTE-HsdS) shows an unreported open form inter-domain orientation of this subunit. Based on the crystal structure of TTE-HsdS and the closed state model of M.EcoKI-M_2_S_1_, we constructed a potential open state model of type I methyltransferase. Mutational studies indicated that two α-helices (aa30-59 and aa466-495) of the TTE-HsdM subunit are important inter-subunit interaction sites in the TTE-M_2_S_1_ complex. DNA binding assays also highlighted the importance of the C-terminal region of TTE-HsdM for DNA binding by the TTE-M_2_S_1_ complex. On the basis of structural analysis, biochemical experiments and previous studies, we propose a dynamic opening and closing mechanism for type I methyltransferase.

## Introduction

Restriction-modification (R-M) systems maintain the integrity of bacterial genomes by cleaving foreign DNA [1]. Four types of R-M enzymes are presently known: I, II, III, and IV [2], [3]. The most complex of the four enzymes is the type I enzyme which is also the first R-M enzyme discovered [4]. Type I R-M enzymes are composed of three different subunits: a specificity subunit (HsdS or S) that recognizes specific DNA sequences, a methylation subunit (HsdM or M) that methylates target adenine bases, and a restriction subunit (HsdR or R) that translocates from the recognition site and cleaves DNA at variable positions [2], [5]. The HsdS subunit consists of two globular domains that correspond to the variable target recognition domains (TRD1 and TRD2) and two conserved regions (CR1 and CR2) that separate the TRDs. The three subunits can assemble into two types of complexes: R_2_M_2_S_1_ with both methyltransferase and restrictase activities, or M_2_S_1_ with only methyltransferase activity [6]. M_2_S_1_ is also the core DNA-binding component of the R-M enzyme [7]. Together, the M_2_S_1_ complex recognizes an asymmetric, bipartite nucleotide target containing two specific regions 3 to 5 bp in length that are separated by nonspecific DNA sequences of 6 to 8 bp [8], [9].

The orientation of the TRDs and the CRs are quite different between the two published structures for the HsdS subunit (Mja-HsdS [10] and Mge-HsdS [6]). The difference in observed structures suggests that domain motion occurs within the HsdS subunit [7], [11], [12]. However, the structural basis of the inter-domain movements has not been established. Domain motion within the HsdS subunit might result in conformational changes and dynamic opening and closing of the whole M_2_S_1_ complex [13]. The electron microscopy (EM) model of M.EcoKI-M_2_S_1_ fits a closed state type I methyltransferase [13], but does not provide clear information about the open state. Crystal structures of Mja-HsdS [10] and Mge-HsdS [6] based on the mode of inter-subunit interactions in the M.EcoKI-M_2_S_1_ EM model and the domain orientation of the HsdS subunit cannot result in an open form of the M_2_S_1_ complex. An open state model of the M_2_S_1_ complex is needed in order to understand the structure of the complex and to model the dynamic opening and closing of the complex. Three dimensional structures of the HsdS subunit with an open form domain-orientation are therefore needed.

The EM model of M.EcoKI-M_2_S_1_ reveals that the N terminal domains of the two HsdM subunits contact each other, while the C terminal domain of the HsdM subunits contact the HsdS subunit [13]. Other studies indicate that the C terminal region of the HsdM subunit is essential for the assembly of the EcoKI methyltransferase [14], while mutation in the N terminal domain of the HsdM subunit reduces the affinity of the enzyme for hemimethylated targets [15], [16]. There are two possible HsdS-HsdM interfaces in HsdS subunit. One possible interface is the connection region between CRs and TRDs [10], [13], [17]. The other possible interface is at a helix-loop structure in the TRDs [13]. Until now, the exact sites of interaction at the HsdM-HsdS and HsdM-HsdM interfaces have not been identified.

We report here the crystal structure of HsdS from *Thermoanaerobacter tengcongensis* in an open form conformation at 1.95 Å resolution. Based on structural comparisons and modelling, we propose a hemi-open state model for the M_2_S_1_ complex. Also, mutational studies were used to reveal the inter-subunit interaction sites of type I methyltransferases from *T. tengcongensis* (TTE-M_2_S_1_). Based on the structural and mutational evidence presented here, we have supposed a dynamic “opening and closing ” way of the M_2_S_1_ complex.

## Materials and Methods

### Cloning and vector construction

The *tte-hsdS* and *tte-hsdM* gene were amplified by PCR from *T. tengcongensis* genomic DNA [18]. The PCR products of *tte-hsdS* (ORF: TTE1545) and *tte-hsdM* (ORF: TTE1547) were cloned into the pET-DUET co-expression vector at cloning sites 1 (with N-terminal His tag) and 2 (without tag) respectively. Based on this co-expression vector of “wild type TTE-HsdS/wild type TTE-HsdM”, we also constructed several co-expression vectors of “wild type TTE-HsdS/mutant TTE-HsdM”. Details of these co-expression vectors are summarized in **Table 1**. An expression vector of TTE-HsdS alone was also constructed by cloning the PCR product of *tte-hsdS* into the pHAT-2 expression vector.

**Table 1:** Co-expression vectors of TTE-HsdS and TTE-HsdM.

Vetors	MCS1	MCS2
petDUET_SM	TTE-HsdS (wild type)	TTE-HsdM (wild type)
petDUET_SM_Δn10_	TTE-HsdS (wild type)	TTE-HsdM (aa001-010 deletion)
petDUET_SM_Δn30_	TTE-HsdS (wild type)	TTE-HsdM (aa001-030 deletion)
petDUET_SM_Δn40_	TTE-HsdS (wild type)	TTE-HsdM (aa001-040 deletion)
petDUET_SM_Δn50_	TTE-HsdS (wild type)	TTE-HsdM (aa001-050 deletion)
petDUET_SM_Δc10_	TTE-HsdS (wild type)	TTE-HsdM (aa498-507 deletion)
petDUET_SM_Δc21_	TTE-HsdS (wild type)	TTE-HsdM (aa487-507 deletion)
petDUET_SM_Δc30_	TTE-HsdS (wild type)	TTE-HsdM (aa478-507 deletion)
petDUET_SM_Δc40_	TTE-HsdS (wild type)	TTE-HsdM (aa468-507 deletion)

### Protein expression and purification

All vectors were transformed into BL21 (DE3) *Escherichia coli* cells. The cells were grown in LB media supplemented with 100 mg/mL ampicillin until they reached log phase growth (OD600 = 0.6). The expression of TTE-HsdS was induced by stimulation with IPTG (0.4 mM) at 28°C for 10 h. Cells were harvested and resuspended in buffer A (20 mM HEPES pH 7.0, 300 mM NaCl, 5% glycerol, 3 mM β-mercaptoethanol) and then lysed by sonication. The lysate was clarified by centrifugation and purified by passage through a nickel-affinity column. A further purification step was then performed using size exclusion chromatography on a Superdex 200 column (Amersham). The purified protein was concentrated to 15 mg/mL for crystallization in buffer B (5 mM HEPES pH 7.0, 300 mM NaCl, 5% glycerol, 1 mM DTT). A number of TTE-HsdS/TTE-HsdM complexes were expressed and purified using the same protocol.

### Crystallization and data collection

Crystals of recombinant TTE-HsdS were grown at 20°C using the hanging-drop, vapor-diffusion method. Drops consisted of 2 µL of protein solution and 2 µL of mother liquor (0.1 M Bis-Tris pH 6.4, 1.16 M (NH_4_)_2_SO_4_). Crystals suitable for X-ray diffraction studies were obtained after 5 days growth. Hg derivatives were obtained using the same protocol as in our previous work [19]. Native and derivative crystals were soaked in 2 M Li_2_SO_4_ for 2 min before data collection and were flash-frozen in liquid nitrogen. Native crystal data were collected on a beamline NW12A (Photon Factory, KEK, Japan). Derivative data were collected on a Rigaku FR-E X-ray generator with a Rigaku R-AXIS IV++ image plate detector. Data were integrated and scaled with HKL2000 [20]. Statistical analysis of the data collected is summarized in **Table 2**.

**Table 2.** Data collection and refinement statistics.

A. *Data collection statistics*		
	Native data	Hg-derivative data
Wavelength (Å)	1.0000	1.5418
Space group	*P*2_1_2_1_2_1_	*P*2_1_2_1_2_1_
Unit cell parameters		
*a* (Å)	60.966	61.438
*b* (Å)	137.681	137.747
*c* (Å)	142.277	142.629
Resolution (Å)	15–1.95 (2.00–1.95)	15–2.25 (2.30–2.25)
No. unique reflections	87,188	58,230
Redundancy	14.4 (14.0)	13.2 (12.6)
*R*_merge_ (%)	5.0 (35.6)	8.4 (48.4)
Completeness (%)	99.9 (100.0)	99.9 (99.6)
*I/*σ (I)	58.3 (8.5)	27.8 (5.3)

B. *Refinement statistics*		
Resolution (Å)	10–1.95	
*R*_work_ (%)	19.8	
*R*_free_ (%)	23.8	
Non-hydrogen atoms		
Protein	6193	
Water	786	
Ligand	75	
r.m.s.d. from ideal		
Bonds length (Å)	0.010	
Bond angles ()	1.25	
Overall average B factor (Å^2^)	36.6	
Ramachandran plot		
Most favored regions (%)	89.9	
Allowed regions (%)	10.1	
Generously allowed regions (%)	0	
Disallowed regions (%)	0	

### Structure determination and refinement

Six mercury sites in each asymmetric unit were determined using SHELXD [21]. After refinement of the heavy atom parameters, the first density map was obtained by SAD phasing using SHARP [22]. Model building was performed with ARP/wARP [23] and COOT [24] at 1.95 Å resolution. Model refinement was performed in CNS [25], and COOT was used for inspection and manual improvement of the model. Within the resolution range of 10–1.95 Å, the native structure was refined to a final R_work_ = 19.8% and R_free_ = 23.8%. Acceptable stereochemistry was confirmed from a Ramachandran plot calculated by PROCHECK [26]. The final model consists of two TTE-HsdS monomers in the asymmetric unit. Residues 327–334 from both subunits are missing. The statistics of the refinement and stereochemistry of the final model are summarized in **Table 2**. The coordinates and structure factors of TTE-HsdS were deposited into RCSB Protein Data Bank with accession code 3OKG.

### DNA binding assay

A non-radioactive electrophoretic mobility shift assay (EMSA) method was used to inspect the DNA binding properties of wild type and mutant TTE-M_2_S_1_ complexes. Linear DNA used in the experiments was from the vector pGEX6p-1 digested with *EcoR*I and *Not*I. The reaction mixture contained 10 mM HEPES at pH 7.0, 300 mM NaCl, 5% glycerol, 1 mM DTT, linear DNA and TTE-M_2_S_1_ (wide type or mutant). The samples were subjected to agarose gel electrophoresis after 1 h incubation at 20°C.

## Results

### Overall structure of TTE-HsdS

**Figure 1:**
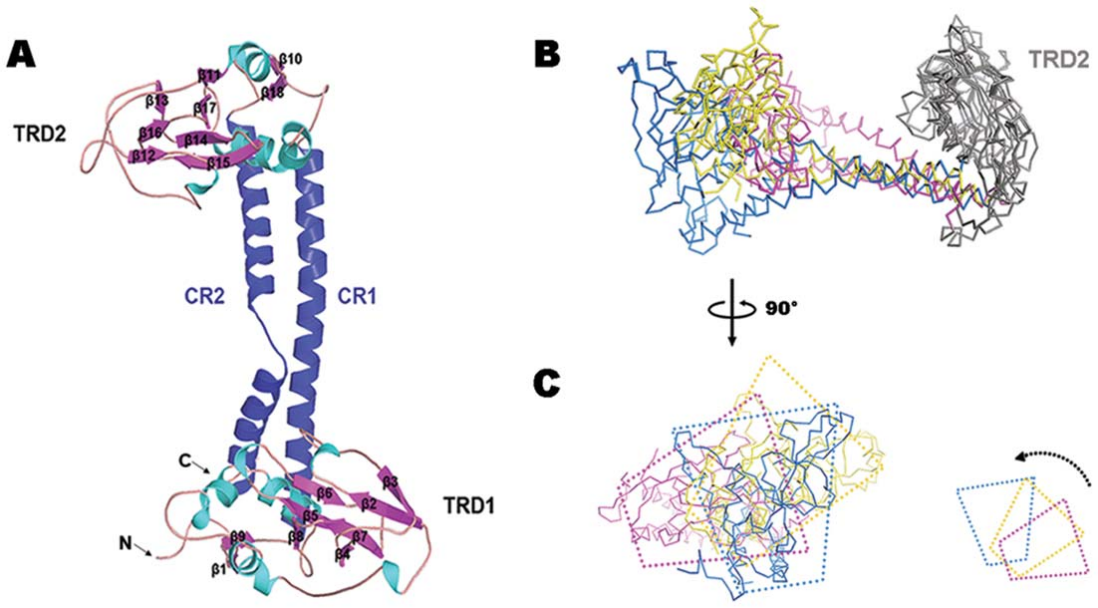
Overall structure of TTE-HsdS and structural superimposition. (A) The monomer fold of TTE-HsdS, revealing a cyclical organization of HsdS subunit. α-helices, β-sheets, and loops in TRDs are colored cyan, magenta, and light pink, respectively. The CRs are colored blue. (B) Superimposition on TRD2 (gray) of TTE-HsdS (blue), Mja-HsdS (yellow) and Mge-HsdS (red). Comparing with Mja-HsdS and Mge-HsdS, the angle and distance between TRD1 and TRD2 of TTE-HsdS are obviously enlarged. (C) The side view of (B). The trapezia indicate the clear differences in domain orientation of TRD1s.

The crystal structure of TTE-HsdS was determined to 1.95 Å resolution by the single wavelength anomalous diffraction method using a mercury derivative (**Table 2**). The monomer structure, containing 398 amino acids, showed four distinct and continuous structural regions: the N-terminal TRD (TRD1, Met1 - Pro159), the central CR (CR1, Leu160 - Phe203), the central TRD (TRD2, Pro204–Pro350) and the C-terminal CR (CR2, Leu351–Leu398) (**Figure 1A**). The overall structure obtained for TTE-HsdS confirmed the expected cyclic topology of the subunit [27].

TRD1 and TRD2 exhibited very similar folds. The three-dimensional structural comparison Z score of the two globular domains given by the DALI PAIRWISE COMPARISON SERVER [28] were 11.1, giving a root mean square deviation (rmsd) of 2.2 Å for 138 structurally equivalent C^α^ atoms. A 2-stranded antiparallel β-sheet was found at the beginning and end of each TRD (β1 and β9 in TRD1, β10 andβ18 in TRD2). The core structure of each TRD consisted of three α-helices and two β-sheets with four and three short strands respectively (β2-β3-β4-β7 and β5-β6-β8 in TRD1, β11-β14-β15-β17 and β12-β13-β16 in TRD2) (**Figure 1A**).

In TTE-HsdS, the CRs were found to be composed of two long antiparallel α-helices, forming a coiled coils motif. The two helices were held together mainly by hydrophobic interactions and four hydrogen bonds (**Figure S1**). A three amino acid loop (Gln375–Glu377) is inserted in the CR2 α-helice. And there is a fifty degree bend in CR2. The angle and distance between TRD1 and TRD2 indicated the open-form domain-orientation of TTE-HsdS (**Figure 1A**).

### Open form conformation of TTE-HsdS

Superposition of the overall structure of TTE-HsdS and two other HsdS subunits (Mja-HsdS and Mge-HsdS) using the DALI PAIRWISE COMPARISON SERVER gave an rmsd of 8.1 Å for 360 structurally equivalent C^α^ atoms and 11.1 Å for 321 structurally equivalent C^α^ atoms (**Figure S2A**). When only the TRDs were superimposed, the following rmsd values were obtained: 3.7 Å for 146 equivalent C^α^ atoms (TRD1s of TTE-HsdS and Mja-HsdS), 3.9 Å for 117 equivalent C^α^ atoms (TRD1s of TTE-HsdS and Mge-HsdS) (**Figure S2B**), 2.1 Å for 124 equivalent C^α^ atoms (TRD2s of TTE-HsdS and Mja-HsdS) and 2.9 Å for 123 equivalent C^α^ atoms (TRD2s of TTE-HsdS and Mge-HsdS) (**Figure S2C**). Although both TRD1 and TRD2 have similar folds in the three HsdS subunits, the overall domain orientation is quite different. Hence, significant domain motion could happen within the HsdS subunit. However, the intrasubunit conformational changes are not well understood. By superimposing the TRD2s of the three HsdS subunits, differences between the TTE-HsdS and the other two HsdS subunits could be described in three ways. Firstly, significant bending and twisting of the CRs occurs within TTE-HsdS, giving rotations of 23.7° and 33.8° when compared to Mja-HsdS and Mge-HsdS (**Figure 1B**). Secondly, the angle and distance between TRD1 and TRD2 in TTE-HsdS is larger than in Mja-HsdS and Mge-HsdS (**Figure 1B**). Thirdly, there is an obvious rotation of TRD1 with respect to TRD2 in TTE-HsdS versus the other two HsdS subunits (**Figure 1C**). By superimposing the CRs of the three HsdS subunits, significant conformational differences are also found in CR2s and TRDs (**Figure S3**). Comparisons among the above structures revealed that the TTE-HsdS subunit is in a relatively open conformation. The proposed HsdS-HsdM interaction sites are located in the connection region of CRs and TRDs and in a helix-loop region in TRDs [10], [13]. Domain motion of HsdS subunits would induce a corresponding movement of HsdM subunits. As a result, the M_2_S_1_ complex is able to undergo conformational changes.

### Potential open state of M_2_S_1_ complex

**Figure 2:**
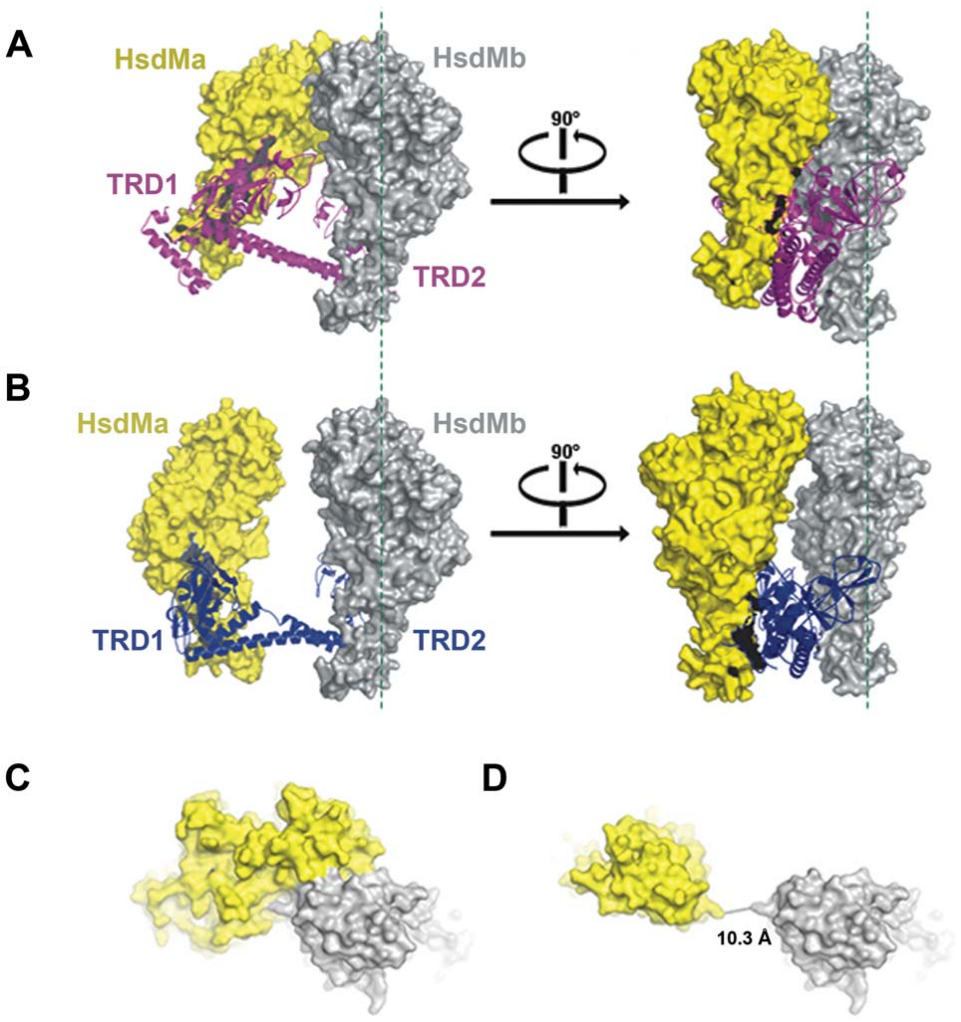
Models of M_2_S_1_ in Closed state and open state. (A) The closed state model of M.EcoKI-M_2_S_1_, showing the HsdM-HsdM interaction via their N-terminal domains. HsdS is shown as a red ribbon. The two HsdMs (in yellow and gray) are shown as surface. (B) The open state model of TTE-M_2_S_1_. Significant bending and twisting occurs in the CRs, moving the N-terminal domains of HsdMs apart. HsdS is shown as a blue ribbon. The two HsdMs (in yellow and gray) are shown as surface. (C) The top view of M.EcoKI-M_2_S_1_ closed state model highlighting the interface between the N-terminal domains of HsdMs. (D) The top view of TTE-M_2_S_1_ open state model highlighting the separated N-terminal domains of HsdMs.

Stable M_2_S_1_ complexes were purified by co-expression of TTE-HsdS and TTE-HsdM in *E.coli*. Expression of TTE-HsdM alone was insoluble. The molecular weight of the protein complex was determined by analytical ultracentrifugation to be 165 kDa, indicating that the protein complex consists of two HsdM subunits (MW: 58.5 kDa) and one HsdS subunit (MW: 46.5 kDa) (**Figure S4**). The open form conformation structure of TTE-HsdS and the closed state model of M.EcoKI-M_2_S_1_ complex (**Figure 2A, C**) were used to construct the open state model of the M_2_S_1_ complex. By means of superimposition and replacement of TRDs, we replaced EcoKI-HsdS in the M.EcoKI-M_2_S_1_ model with TTE-HsdS to generate the model of TTE-M_2_S_1_ (one TTE-HsdS and two EcoKI-HsdM) (**Figure 2B, D** **and Figure S5**). As expected, the TTE-M_2_S_1_ model revealed a completely different open state conformation than the M.EcoKI-M_2_S_1_ model. In addition, the open state TTE-M_2_S_1_ model clearly showed that the clamp-like closed state complex converts to the open state by movement of the N-terminal domains of the HsdM subunits. By using the same protocol, crystal structures of Mja-HsdS and Mge-HsdS can not result in a reasonable open form of the M_2_S_1_ complex (**Figure S6**).

### Inter-subunit interactions of TTE-M_2_S_1_

**Figure 3:**
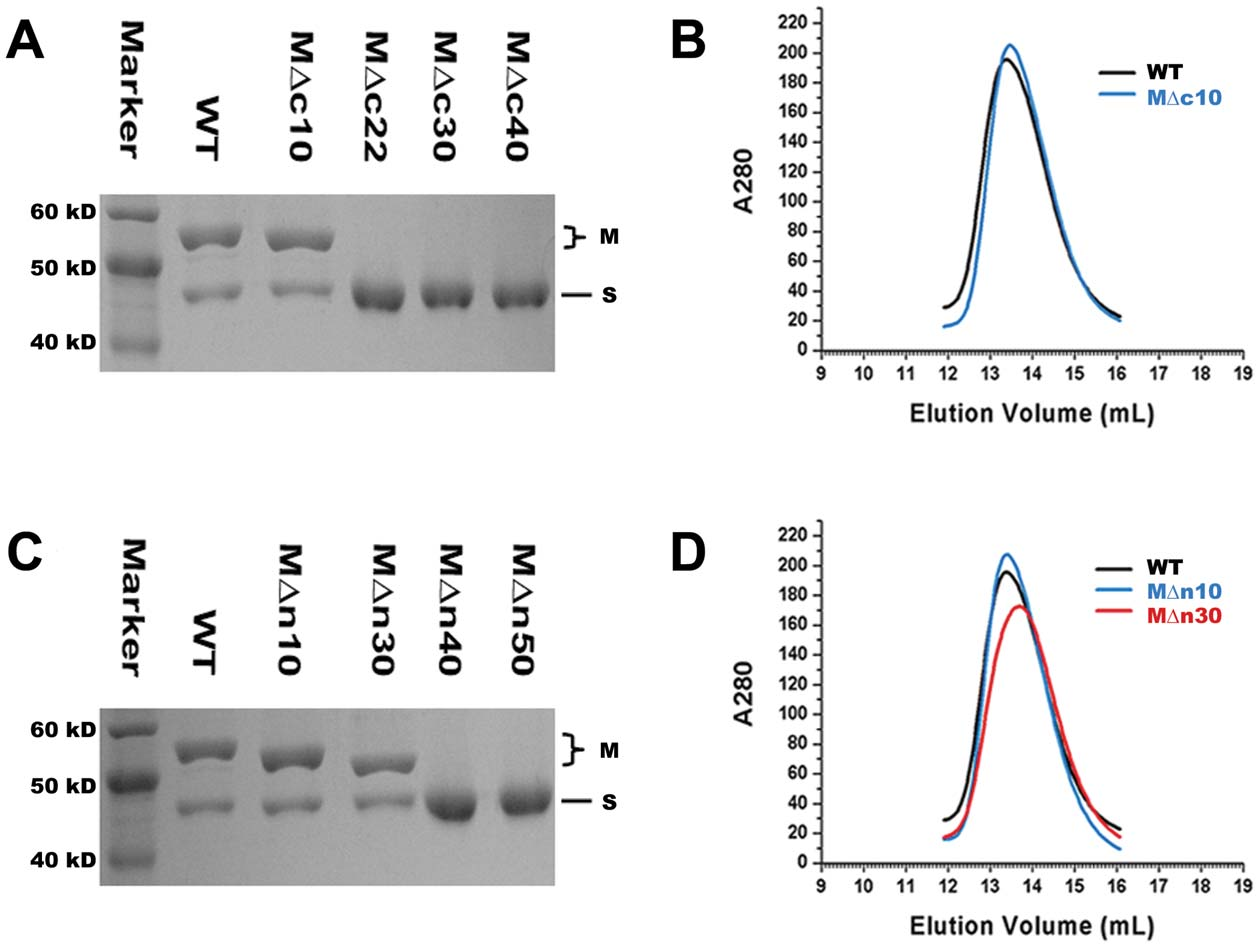
Mutational assays of TTE-M_2_S_1_. (A) SDS-PAGE of the co-purification of wild type HsdS and HsdM mutants with C-terminal deletion. Only MΔc10 can be co-purified with HsdS subunit. (B) Gel filtration analysis of TTE-M_2_S_1_ and TTE-HsdMΔc10_2_S_1_, showing the similar aggregation state between them. (C) SDS-PAGE of the co-purification of wild type HsdS and HsdM mutants with N-terminal deletion. Only MΔn10 and MΔn30 can be co-purified with HsdS subunit. (D) Gel filtration analysis of TTE-M_2_S_1_, TTE-HsdMΔn10_2_S_1_ and TTE-HsdMΔn30_2_S_1_, showing the similar aggregation state among them.

Type I methyltransferase will remain in the closed state when no DNA is entering or leaving the complex [13]. The M.EcoKI-M_2_S_1_ EM model shows that the HsdM subunit C-terminal region contacts the HsdS subunit while the N-terminal regions of the HsdM subunits contact each other. A series of mutation assays were designed in order to confirm these proposed contact regions and identify the specific interaction sites. Firstly, we constructed four co-expression vectors consisting of wild type TTE-HsdS and different TTE-HsdM C-terminal deleted mutants (petDUET_SMΔc10, petDUET_SMΔc21, petDUET_SMΔc30 and petDUET_SMΔc40) (**Table 1**). Further purification experiments showed that TTE-HsdMΔc10 (Δ498–507) can form stable complex with wild type TTE-HsdS but TTE-HsdMΔc21 (Δ487–507), TTE-HsdMΔc30 (Δ478–507) and TTE-HsdMΔc40 (Δ468–507) cannot form stable complexes (**Figure 3A**). Size exclusion chromatography revealed that the complex formed by TTE-HsdMΔc10 and TTE-HsdS has the same subunit composition as the wild type complex **(** **Figure 3B**), indicating that the HsdM-HsdS interaction sites are intact in TTE-HsdMΔc10. Residues 466–495 in TTE-HsdM are predicted to form an α-helix, while predictions for TTE-HsdMΔc21, TTE-HsdMΔc30 and TTE-HsdMΔc40 lack this secondary structure element. Therefore, the α-helix in the C-terminal region of TTE-HsdM is an important HsdM-HsdS interaction site.

Four co-expression vectors were also constructed of wild type TTE-HsdS with different TTE-HsdM N-terminal deleted mutants (petDUET_SMΔn10, petDUET_SMΔn30, petDUET_SMΔn40 and petDUET_SMΔn50) (**Table 2**). Purification results showed that only TTE-HsdMΔn10 (Δ1–10) and TTE-HsdMΔn30 (Δ1–30) can form stable complexes with TTE-HsdS (**Figure 3C**). Also, the subunit composition of the two mutant complexes is the same as the wild type complex (**Figure 3D**). These results clearly show that the deletion of residues 1–30 of TTE-HsdM does not affect HsdM-HsdM interactions, but that the additional deletion of residues 30–40 or residues 30–50 will disrupt the interaction (**Figure 3C**). The secondary structure prediction shows that residues 30–59 in TTE-HsdM form an α-helix. Damage to this α-helix structure, as in the Δ1–40 and Δ1–50 mutants disrupts HsdM-HsdM interactions and undermines the stability of the TTE-M_2_S_1_ complex.

### Interaction of DNA and TTE-M_2_S_1_

**Figure 4:**
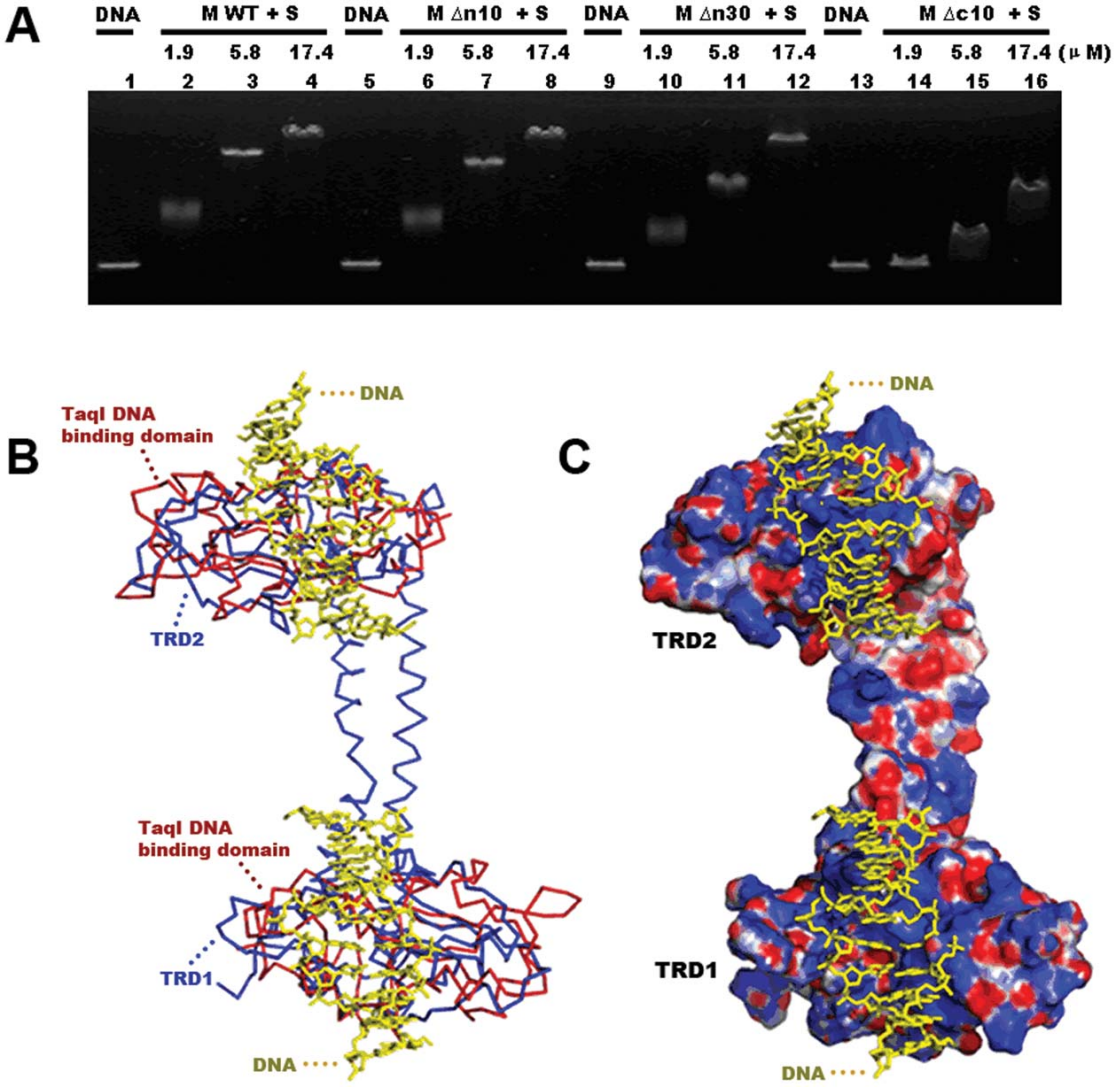
Interactions of DNA and TTE-M_2_S_1_. (A) EMSA assays of wild type TTE-M_2_S_1_ and its mutants. TTE-MΔn10_2_S_1_ and TTE-MΔn30_2_S_1_ have the similar DNA binding affinity with wild type TTE-M_2_S_1_, but the DNA binding affinity of TTE-MΔc10_2_S_1_ is much weaker. (B) Superposition of the DNA binding domain (red ribbon) of TaqI Mtase onto the two TRDs of TTE-HsdS (blue ribbon). The DNA molecules are shown as yellow stick. (C) Electrostatic potential maps of the surface of TTE-HsdS and the DNA binding model. Positive potential is colored blue, neutral potential is colored gray, and negative potential is colored red.

Until now, there has been no DNA binding information for *T. tengcongensis* Type I methyltransferase M_2_S_1_ complex. Results of our EMSA assay showed that the mixture of linear vector DNA and wild type TTE-M_2_S_1_ was less mobile than free DNA, an effect that was more obvious as the concentration of protein complex was increased (**Figure 4A**). This indicates that TTE-M_2_S_1_ can non-specifically bind to linear vector DNA. Unspecific binding with linear DNA is also found with three of the mutant M_2_S_1_ complexes (TTE-MΔn10_2_S_1_, TTE-MΔn30_2_S_1_ and TTE-MΔc10_2_S_1_). TTE-MΔn10_2_S_1_ and TTE-MΔn30_2_S_1_ had similar linear DNA binding affinities as wild type TTE-M_2_S_1_ (**Figure 4A**), indicating that the deletion of residues 1–30 from the N-terminal region of TTE-HsdM does not affect the interaction of the complex with DNA. However, the DNA binding affinity of TTE-MΔc10_2_S_1_ was weaker than wild type complex DNA binding affinity (**Figure 4A**). This shows the importance of the C-terminal region of the TTE-HsdM subunits for M_2_S_1_ complex binding with linear DNA.

In order to identify the TTE-HsdS DNA binding sites, TRD1 and TRD2 were entered into the DALI server [29] to search for structurally related proteins. The search results showed that the DNA binding domain of *Taq*I-Mtase [30] has folds similar to TRD1 and TRD2 with rmsd values of 3.1 Å and 3.2 Å respectively. Putative DNA binding regions in the TRDs were immediately identified from the superposition of the DNA binding domain from *Taq*I-Mtase and the TRDs (**Figure 4B**). Conspicuous sections of positively charged residues are found in the DNA binding regions (**Figure 4C**). Through comparison with the DNA binding domain of *Taq*I-Mtase, residues in several equivalent loops of TRDs were found to be involved in DNA binding (TRD1: Asp41-Ser43, Pro64-Arg66, Thr81-Arg82, Ser101-Thr102 and Ser141-Ala144; TRD2: Ser230-Ser233, Gly248-Lys249, Arg280-Ala281, Arg297-Gly298 and Thr332-Asn334), which is consistent with the results of random point mutagenesis studies in EcoKI [31], [32].

## Discussion

Compared to the EcoKI-HsdS computational model, significant bending and twisting of the CRs in TTE-HsdS crystal structure enlarges the angle and distance between the TRDs and also shows a small range of rotation between the TRDs. Changes of domain orientation in the HsdS subunit are accompanied by movement of the HsdM subunits that interact with the CRs and TRDs. Interactions of N-terminal domains of the HsdM subunits are also lost. We assume that this series of conformational changes reveals the structural basis mediating the conversion between closed and open states. In our TTE-M_2_S_1_ model, the minimum distance between the N-terminal domains of the two HsdM subunits is about 10.3 Å, which is close to but not sufficient to allow the passage of DNA. Therefore, the TTE-M_2_S_1_ model might reflect an intermediate state which is near to the fully open state. Obviously, this open state model is not very sophisticated for lacking of direct experimental evidence, but it implicates a reasonable picture of the flexible clamp-like enzyme.

Our mutational experiments indicated that α-helices formed by residues 30–59 and 466–495 of the HsdM subunits are important sites for HsdM-HsdM and HsdM-HsdS interactions, respectively. Damage to either region will disrupt the corresponding interaction and affect assembly of the M_2_S_1_ complex. However, the N-terminal domains of the HsdM subunits move apart to open the clamp-like complex when DNA is entering or leaving the complex. Target DNA might act as a bridge connecting the N-terminal domains of the HsdM subunits, thereby stabilizing the complex. Therefore, target DNA could facilitate the conversion of the complex from closed to open state.

The computational M.EcoKI-M_2_S_1_ and TTE-M_2_S_1_ models represent the potential closed and open states of type I methyltransferase respectively (Figure 2). These models also indicate that the N-terminal domains of HsdM subunits will move apart from each other during the transition from the closed to open state. According to the results of the EMSA assay and mutational experiments, the target DNA will likely contact the N-terminal domains of the two HsdM subunits to stabilize the complex when DNA is entering or leaving the complex. According to these observations, we speculated a possible “open-close-open” mechanism on the methylation of the target DNA by the M_2_S_1_ complex. Without DNA binding, the M_2_S_1_ is in a closed state [13]. But when target DNA is present, the HsdM-HsdM interaction opens to let the DNA in. Then, the M_2_S_1_ will return to a closed state [13] and the DNA will be methylated. Once the DNA has been methylated, the M_2_S_1_ complex will transit to an open state to release the target DNA and return to the closed state.

In summary, the crystal structure of TTE-HsdS shows an open form domain-orientation. Conformational differences among TTE-HsdS, Mja-HsdS and Mge-HsdS suggest intra-subunit movements within the HsdS subunit. The structural character of this domain motion was discussed via structural comparison. The potential open state model of the M_2_S_1_ complex was proposed based on the structure of TTE-HsdS. Combined with the M.EcoKI-M_2_S_1_ closed model, the open state M_2_S_1_ model reveals the structural basis of dynamic opening and closing of this clamp-like enzyme. Mutational studies identified two α-helices in the N- and C-terminal regions of the HsdM subunit that play crucial roles in inter-subunit interactions. In addition, DNA binding assays also showed the importance of the HsdM C-terminal region for DNA binding by the M_2_S_1_ complex. Based on the work carried out here and in previous studies, we supposed a potential mechanism for the dynamic opening and closing of type I methyltransferase. Notably, many details regarding the hypothesis are still uncertain. More concrete structures and relative investigations are needed for confirmation of this mechanism.

## Supporting Information

Figure S1 **Interactions between the CRs.** Hydrophobic residues are shown in dots model. Residues formed H-bonds (shown in dashes) are shown in stick model.
(TIF)

Figure S2 **Superimposition of HsdS Structures.** (A) Stereo view of the overall superimposition of the TTE-HsdS (blue), Mja-HsdS (yellow) and Mge-HsdS (red) structures. (B) Stereo view of superimposition of TRD1s. (C) Stereo view of superposition of TRD2s.
(TIF)

Figure S3 **Superimposition of CRs.** Superimposition of CRs of the TTE-HsdS (blue), Mja-HsdS (yellow) and Mge-HsdS (red) structures.
(TIF)

Figure S4 **Analytical ultracentrifugation analysis.** The molecular weight of the protein complex was determined to be 165 kD, indicating that the protein complex consists of two TTE-HsdM subunits (MW:58.5 kD) and one TTE-HsdS subunit (MW:46.5 kD).
(TIF)

Figure S5 **The modeling procedure of TTE-M2S1.** EcoKI-HsdS (red) and TTE-HsdS (blue) are shown in cartoon model. HsdM subunits are shown in surface model.
(TIF)

Figure S6 **The potential states of M_2_S_1_ complex.** The potential states of M_2_S_1_ complex based on the conformations of Mge-HsdS (A), Mja-HsdS (B), EcoKI EM model (C) and TTE-HsdS (D). HsdS subunits and HsdM subunits are shown in cartoon model and surface model respectively.
(TIF)
