# Supplementary figures and images for: Structure of HsdS Subunit from Thermoanaerobacter tengcongensis Sheds Lights on Mechanism of Dynamic Opening and Closing of Type I Methyltransferase
Source: PLoS One. 2011 Mar 2;6(3):e17346. doi: 10.1371/journal.pone.0017346 (PMC3047542; doi:10.1371/journal.pone.0017346)

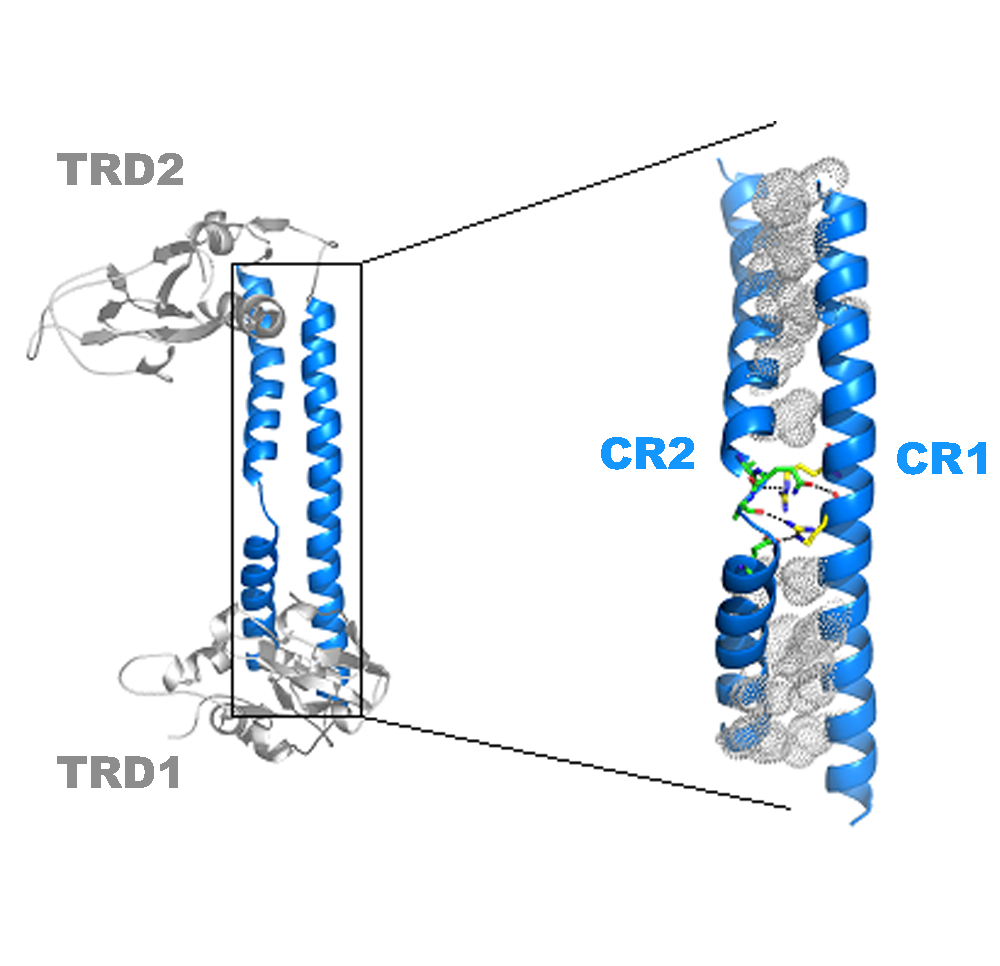

Supplement: Figure S1 — Interactions between the CRs. Hydrophobic residues are shown in dots model. Residues formed H-bonds (shown in dashes) are shown in stick model. (TIF) [file pone.0017346.s001.tif]

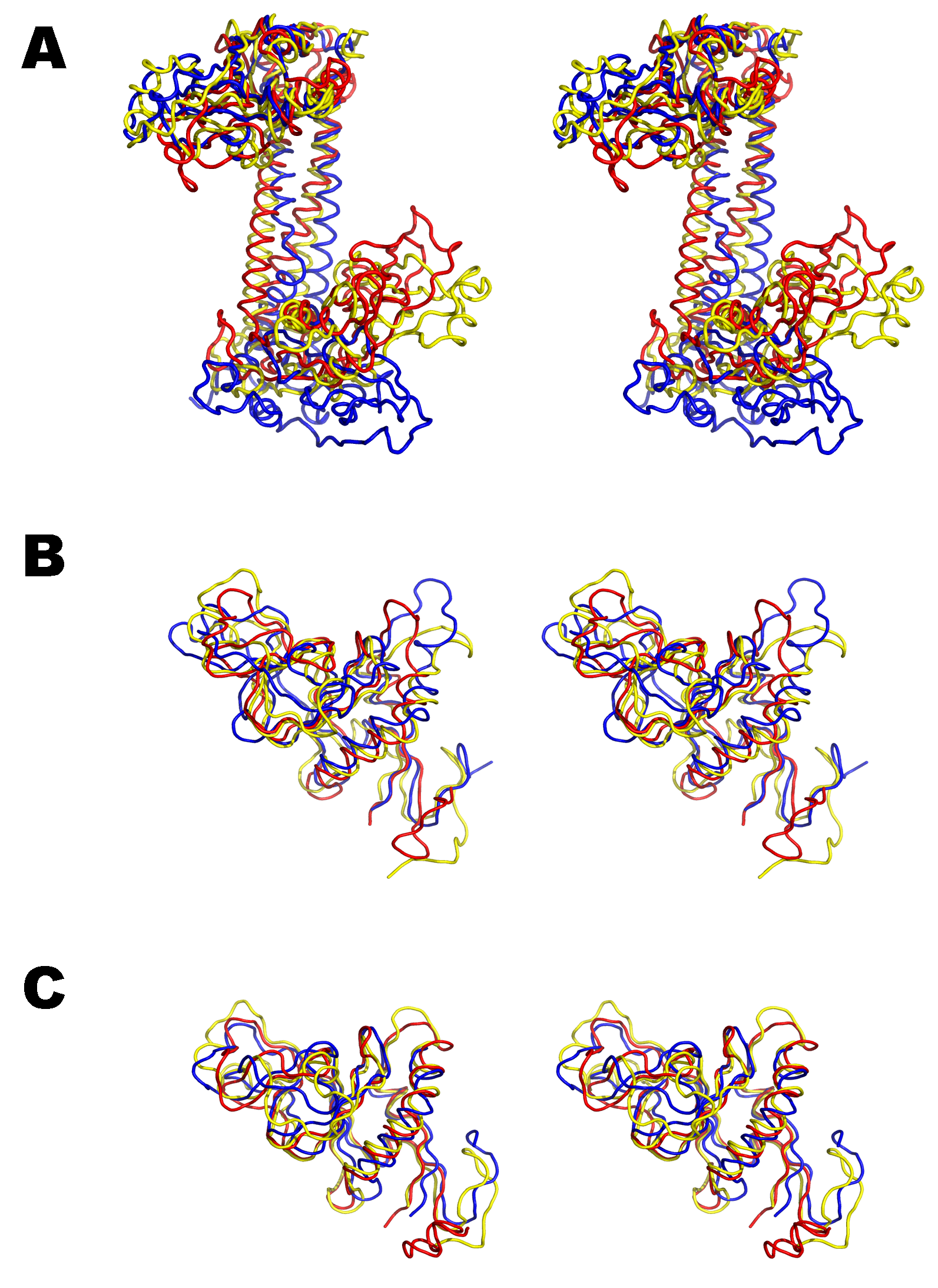

Supplement: Figure S2 — Superimposition of HsdS Structures. (A) Stereo view of the overall superimposition of the TTE-HsdS (blue), Mja-HsdS (yellow) and Mge-HsdS (red) structures. (B) Stereo view of superimposition of TRD1s. (C) Stereo view of superposition of TRD2s. (TIF) [file pone.0017346.s002.tif]

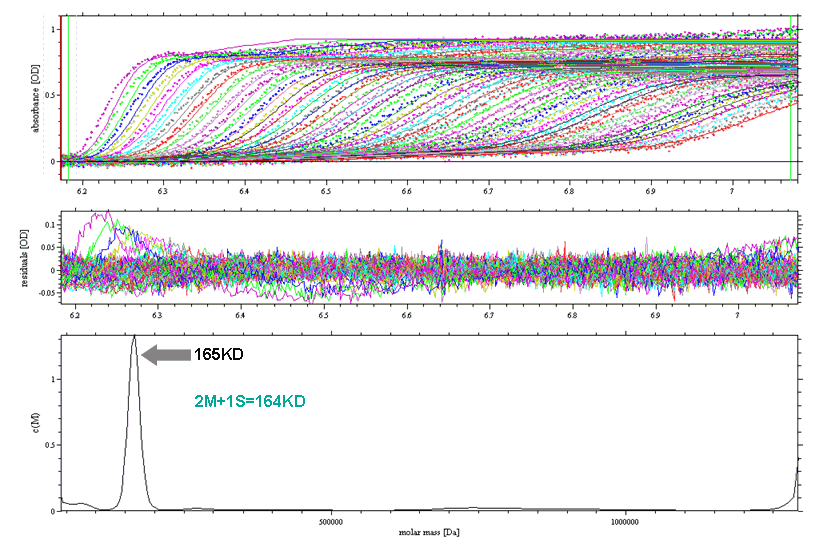

Supplement: Figure S4 — Analytical ultracentrifugation analysis. The molecular weight of the protein complex was determined to be 165 kD, indicating that the protein complex consists of two TTE-HsdM subunits (MW:58.5 kD) and one TTE-HsdS subunit (MW:46.5 kD). (TIF) [file pone.0017346.s004.tif]

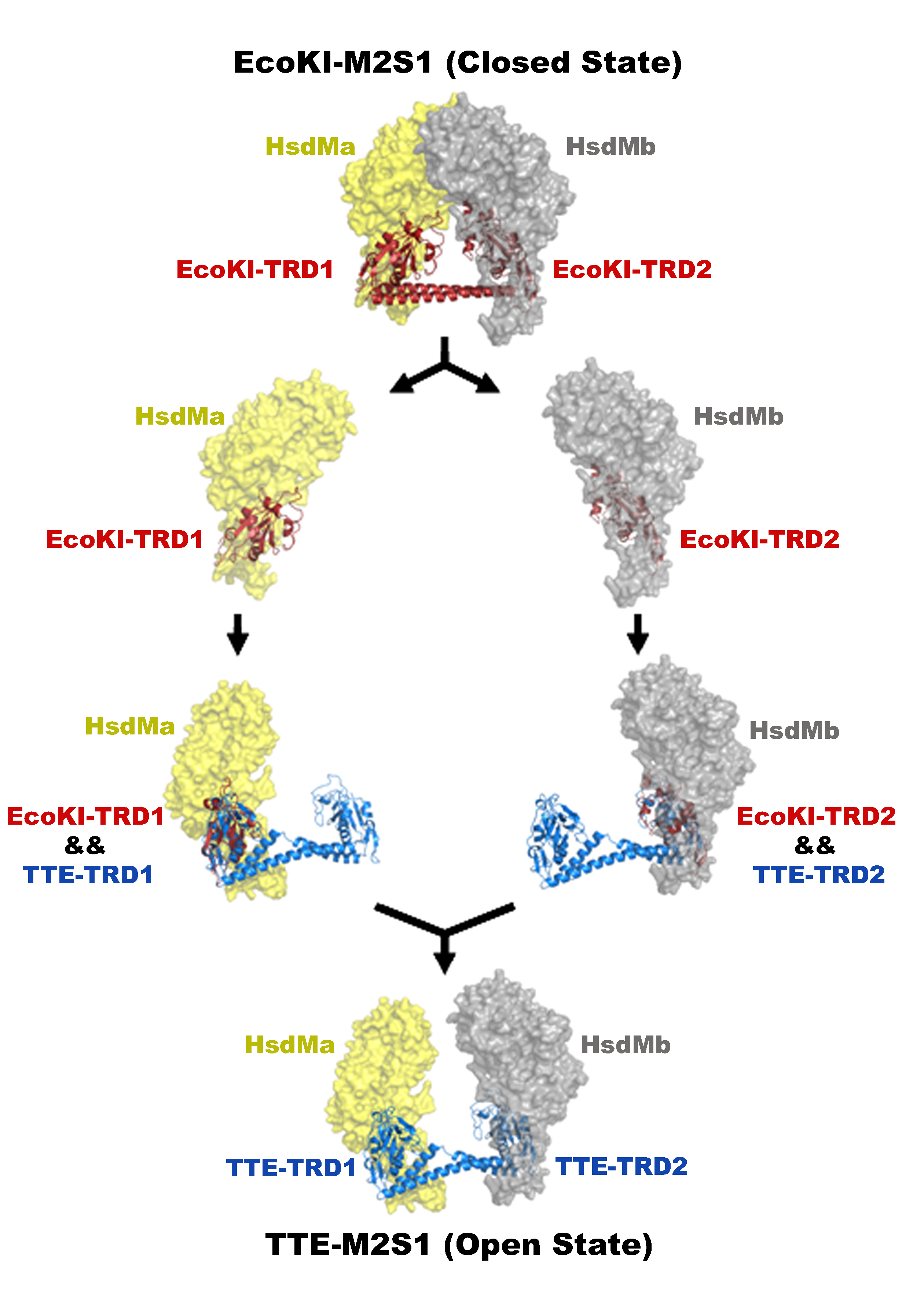

Supplement: Figure S5 — The modeling procedure of TTE-M2S1. EcoKI-HsdS (red) and TTE-HsdS (blue) are shown in cartoon model. HsdM subunits are shown in surface model. (TIF) [file pone.0017346.s005.tif]

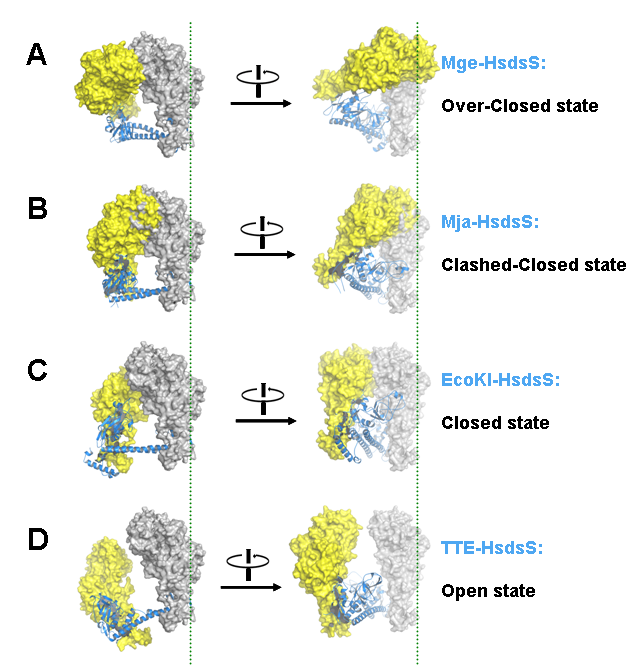

Supplement: Figure S6 — The potential states of M2S1 complex. The potential states of M2S1 complex based on the conformations of Mge-HsdS (A), Mja-HsdS (B), EcoKI EM model (C) and TTE-HsdS (D). HsdS subunits and HsdM subunits are shown in cartoon model and surface model respectively. (TIF) [file pone.0017346.s006.tif]
